# Digital Impressions for Customized Post-and-Core Restorations in Deep and Narrow Canals: A Case Report Utilizing Scan Posts

**DOI:** 10.1155/crid/4939176

**Published:** 2025-12-01

**Authors:** Ling Lin, Pingping Cai, Zhifeng Zheng, Zhiqiang Zheng, Jie Lin

**Affiliations:** ^1^Fujian Key Laboratory of Oral Diseases, School and Hospital of Stomatology, Fujian Medical University, Fuzhou, Fujian, China; ^2^Laboratory Room, School and Hospital of Stomatology, Fujian Medical University, Fuzhou, Fujian, China; ^3^Department of Crown and Bridge, School of Life Dentistry at Tokyo, The Nippon Dental University, Tokyo, Japan

**Keywords:** CAD/CAM, dentistry, digital impression, intraoral scanner, post-and-core, scan post

## Abstract

**Background:**

Traditional impression techniques for customized post-and-core restorations often encounter challenges such as bubble and void formation, requiring a high level of skill from practitioners. Recent advancements in digital technology, particularly intraoral scanners (IOSs), have improved restoration processes, though capturing post spaces with IOS remains problematic due to their deep and narrow nature.

**Case Presentation:**

This report details the use of digital impressions for post-and-core restorations in both anterior and posterior teeth. By designing a scan post that conforms to the shape of the post space, accurate data capture of the post space is achieved. The integration of IOS with computer-aided design/computer-aided manufacturing (CAD/CAM) technology enables the creation of post-and-core restorations that precisely fit the root canal morphology. This approach results in a thin and evenly distributed cement layer, thereby reducing the risk of tooth fracture.

**Conclusion:**

This technique effectively overcomes the limitations of traditional silicone impressions for customized post-and-core restorations. It offers a viable restoration method for single-rooted teeth and addresses challenges related to acquiring impressions for split cast post-and-cores. However, further laboratory research and clinical trials are necessary to thoroughly evaluate the accuracy and reliability of this method.

## 1. Introduction

Endodontically treated teeth are generally considered more fragile and prone to fracture [1]. This increased susceptibility is not only due to significant tissue loss from trauma or dental caries but also stems from the endodontic treatment itself [2]. To mitigate the risk of fracture, endodontically treated teeth often require post-and-core restorations [3].

Advancements in dental materials science have led to the growing preference for prefabricated fiber posts among practitioners. Hawthan et al. [3] found in a preference survey that most dentists favor prefabricated fiber-reinforced composite (FRC) posts. The advantages of fiber posts include ease of manipulation, superior aesthetic performance, and an elastic modulus comparable to that of dentine, which enhances stress distribution across the tooth [4]. However, prefabricated posts often struggle to adapt well to the unique shape of the post space, particularly in elliptical or flared canals [5]. This typically results in a thick resin cement layer, which can contribute to debonding of the posts or even tooth fracture [6]. In contrast, customized post-and-core restorations offer a more effective solution for extensively damaged teeth, wide root canals, and molars subjected to excessive biting forces. Furthermore, in root canals with atypical morphology or excessive width, conventional prefabricated fiber posts may struggle to achieve ideal adaptation [7]. In such scenarios, clinicians may resort to modified techniques, such as customizing fiber posts (by directly building up a composite resin post within the canal) or utilizing auxiliary fiber posts (using multiple prefabricated posts to fill a wide post space). These methods aim to improve post adaptation and reduce the resin cement layer. However, these techniques remain heavily operator-dependent and can be technically challenging and time-consuming. The advent of CAD/CAM technology offers a novel digital solution for fabricating highly accurate customized post-and-cores, potentially overcoming the limitations of these conventional approaches [8].

With the advancement of CAD/CAM technology, customized post-and-cores can now be fabricated using a digital workflow [9]. This digital approach offers several advantages over traditional techniques, including increased efficiency, reduced chairside time, and lower material costs. Research indicates that digital impressions of prepared teeth for single crowns provide better marginal accuracy compared to traditional elastomeric impressions [10]. Recent advancements in digital workflows have shown growing applications in post-and-core restorations. However, as highlighted by Jardim and Lemos [11] in their scoping review, the current literature remains dominated by in vitro studies (13 out of 16 included studies), with a notable lack of high-quality clinical evidence. Significant variations in scanning depth, light conditions, scan post usage, and operator experience contribute to inconsistent outcomes [12], underscoring the urgent need for standardized protocols and further clinical validation [13]. This case report addresses this gap by systematically implementing scan post-assisted digital impressions under controlled clinical conditions, providing practical insights and supporting the transition toward more reproducible and clinically validated digital workflows for post-and-core restorations.

Elter et al. [14] explored the use of IOS for capturing post spaces of varying lengths and found that scanning accuracy was affected by the depth of the post space. Specifically, IOS can effectively capture post spaces with a depth of 14 mm or less and a minimum diameter of 2.2 mm. For post spaces exceeding 14 mm in depth, the narrow and deep nature of the root canal poses challenges for obtaining a complete digital impression. To address this, it is advantageous to use a compatible scan post within the post space to facilitate accurate scanning.

This article discusses the application of scan posts in the fabrication of customized post-and-cores for both anterior and posterior teeth, highlighting the benefits and practical considerations of this digital approach. The protocol was approved by the Research Ethics Committee of the School and Hospital of Stomatology, Fujian Medical University (Approval No. FMUSHS_REC_2024/83).

## 2. Case Presentation 1

**Figure 1:**
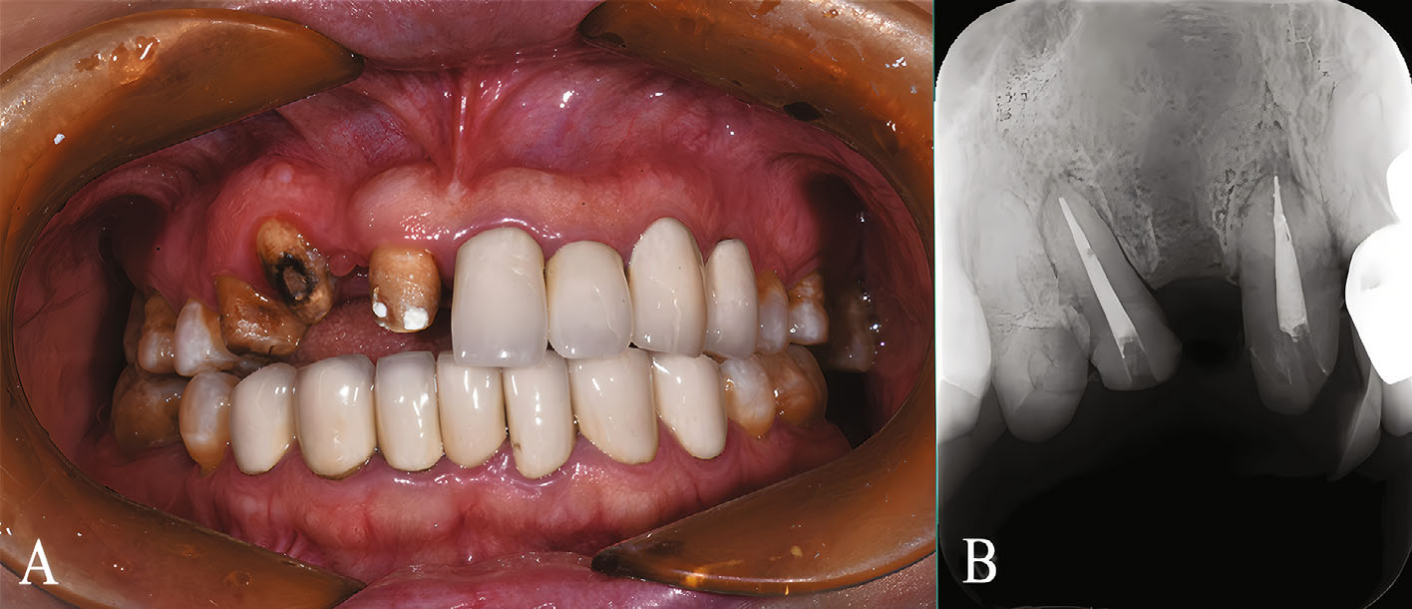
Preoperative clinical and radiographic examination. (A) Frontal intraoral view showing the clinical condition prior to restoration, including existing restorations, tooth structure, and soft tissue architecture. (B) Preoperative periapical radiograph confirming the completion of root canal treatment and revealing the periapical bone condition and root morphology.

A 46-year-old female patient presented with a chief complaint of detachment of a right maxillary crown and desired aesthetic improvement (Figure 1A). Clinical and radiographic examination revealed that root canal treatment had been completed on the central incisor and canine, while the first premolar showed no significant pulpal abnormalities (Figure 1B). Intraoral assessment identified multiple issues: The central incisor had a 5-mm crown height with lingual filling material; the lateral incisor was missing; the canine exhibited gingival recession on the labial shoulder and contained existing fillings with partially decayed tissue on its labial surface. The first premolar also displayed a preexisting tooth preparation morphology.

The treatment was aimed at restoring both function and aesthetics, with particular emphasis on preserving remaining tooth structure and ensuring long-term stability. The restorations needed to be biomechanically sustainable, especially given the compromised condition of the canine and the missing lateral incisor. Additionally, the plan incorporated a digital workflow to enhance precision, improve efficiency, and achieve optimal marginal adaptation of the final prostheses.

For the central incisor, a prefabricated FRC restoration was considered suitable due to its adequate crown height and favorable biomechanical conditions. In contrast, the canine—with its reduced crown height and wider apical root canal—presented a significant challenge, as using a standard FRC restoration could introduce torsional forces and lead to the debonding of the post core. Therefore, a customized post-and-core restoration was selected to better dissipate stresses and improve retention. To restore the entire quadrant, a porcelain-fused-to-metal (PFM) fixed dental prosthesis extending from the central incisor to the first premolar was planned, which would provide functional durability and aesthetic integration. A fully digital workflow was employed throughout the process to leverage the benefits of intraoral scanning and CAD/CAM fabrication.

The post spaces in Teeth 11 and 13 were prepared using #1 through #3 post drills (RelyX Fiber Post Drill, 3 M ESPE, St. Paul, MN, United States). A portion of the gutta-percha was removed, leaving a 3–5-mm apical seal. The treatment approach for Teeth 21 and 23 differed from that of Teeth 11 and 13. For Tooth 11, a No. 3 fiber post (RelyX Fiber Post, 3 M ESPE) was placed and secured with resin cement (RelyX Unicem, 3 M ESPE). For Tooth 23, the new digital impression technique with a scan post was employed, followed by the fabrication and cementation of a customized metal post-and-core.

**Figure 2:**
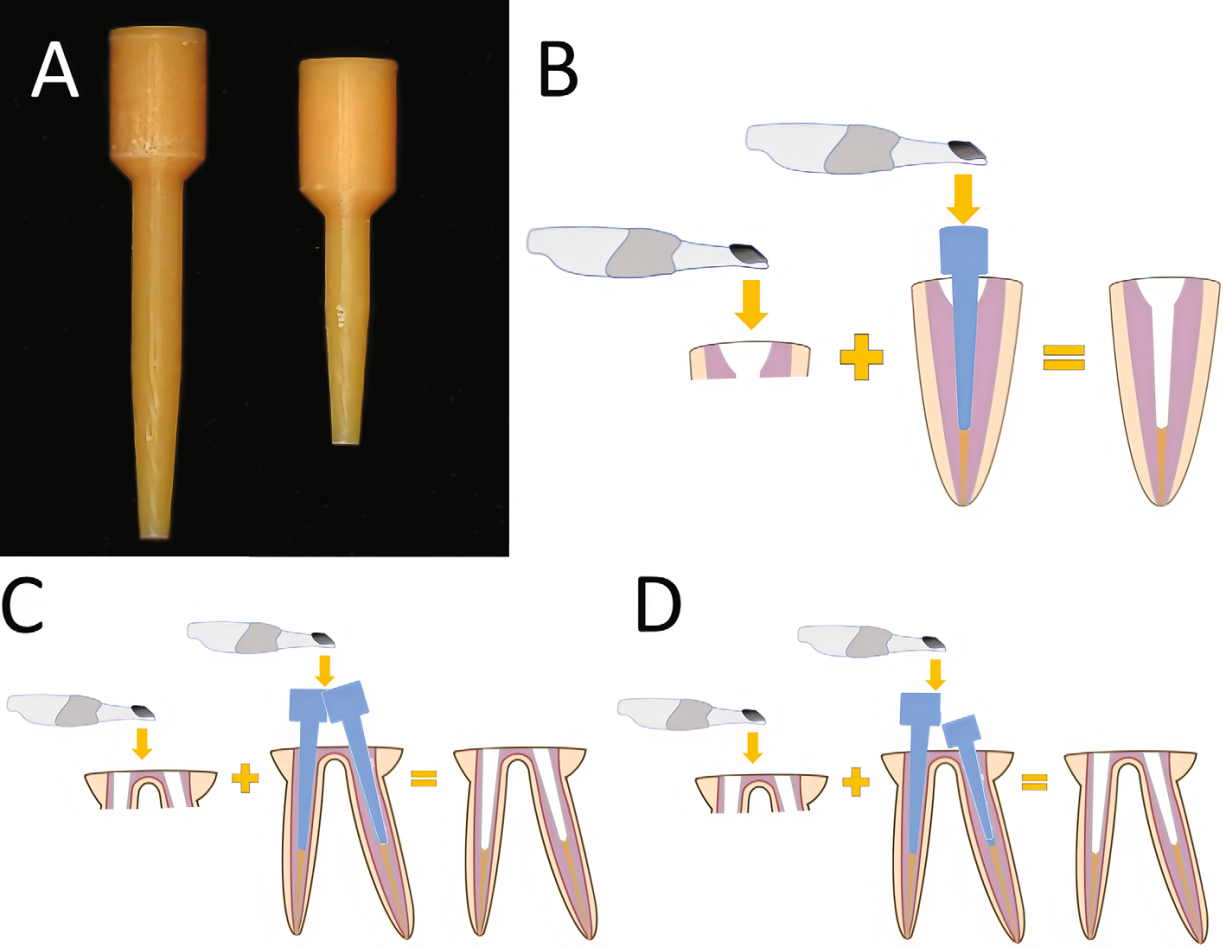
Scan posts and schematic diagram of their application. (A) Fabrication of long and short scan posts using a 3D printer. (B) Schematic illustration of single scan post application: The intraoral scanner first captures the abutment and coronal portion of the post space and then records the cylindrical head of the scan post. These datasets are superimposed in CAD software to generate a complete representation of the post space, following a principle analogous to the impression-taking workflow for dental implants. (C, D) Schematic demonstration of two scan posts used simultaneously. Based on clinical experience, when two scan posts of identical length are used (C), interference during seating may occur. As shown in (D), employing one long and one short scan post significantly reduces the potential for interference.

To accommodate different clinical scenarios, both a long scan post and a short scan post, compatible with the drills, were fabricated using a three-dimensional (3D) printer (Ruiyi DLP1080EA, Han's Laser, Shenzhen, China) (Figure 2). The long and short scan posts used in this study were not provided by the IOS system for post space scanning. Instead, they were custom-designed by first digitally scanning a No. Three RelyX Fiber Post (3 M ESPE) to replicate its dimensions, then adding an enlarged head design to optimize visibility and scanning efficiency, before finally being 3D-printed. The scan posts were fabricated from a photopolymer resin using digital light processing 3D printing technology (Time To Peak, GV-Model 1, Beijing, China). This material was selected for its ability to produce high-resolution, rigid structures with a smooth surface finish, which is essential for achieving accurate optical scanning. Although an alternative method involving physical replication of the entire root canal configuration followed by scanning was considered, this approach was deemed less time-efficient for clinical practice due to the need for patient-specific reproductions. Our goal was to develop a more universal and clinically practical solution. This technique combines the IOS's accurate capture of the supragingival tooth structure with the predefined subgingival geometry provided by the scan post, thereby generating a complete digital impression for the fabrication of the customized post-and-core (Figures 2B, 2C, and 2D). In Case 1, only the long scan post was utilized. In Case 2, however, the two root canals of the posterior tooth were angled. To prevent interference between the two scan posts during simultaneous placement, one long and one short scan post were used.

**Figure 3:**
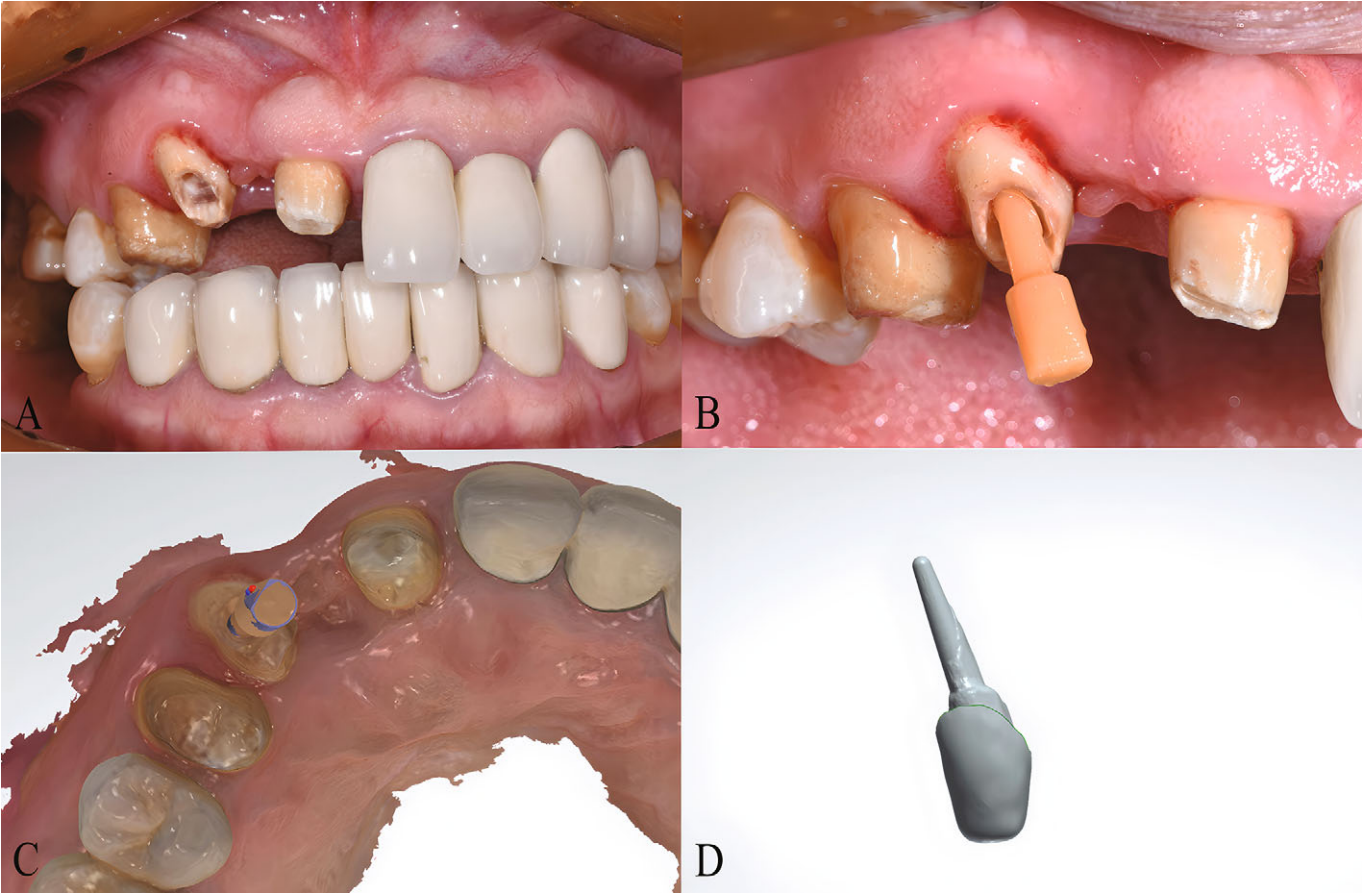
Digital workflow for post space impression using a custom scan post. (A) Initial intraoral scan of the prepared tooth without the scan post, capturing the coronal morphology and post space entrance. (B) Scan with the custom scan post in place, providing geometric reference for the subgingival portion of the post space. (C) CAD software view showing the virtual alignment of the scan post within the post space, ensuring precise dimensional transfer. (D) Designed post-and-core restoration based on the integrated digital data, demonstrating adaptation to the root canal morphology and coronal emergence profile.

The digital workflow was implemented following a standardized protocol. Prior to scanning, the TRIOS 3 IOS (3Shape, Copenhagen, Denmark) was calibrated according to the manufacturer's guidelines. Tooth 13 was scanned with an IOS (TRIOS 3D Intraoral Scanner, 3Shape, Copenhagen, Denmark) first without the scan post (Figure 3A), and then with the scan post in place (Figure 3B). This approach ensured comprehensive data acquisition of the post space, ferrule height, and full digital impressions of both the upper and lower arches, including bite registration. The collected data was then transferred to CAD software (Figure 3C). Throughout the scanning and subsequent bonding procedures, moisture control was maintained through conventional isolation techniques, including rubber dam application and cotton rolls with high-volume suction, ensuring optimal operating field conditions. This protocol was developed based on our previous methodological study [15].

Based on this data, a customized post-and-core was designed (Figure 3D). A virtual cement space of 40 *μ*m was configured in the CAD software to accommodate the resin cement layer. A wax pattern was subsequently milled using a CAM machine (ZENO 4030 M1+, imes-icore GmbH, Eiterfeld, Hesse, Germany). Finally, a cobalt–chromium (Co-Cr) post-and-core (15088-1, DFS-Diamon, Riedenburg, Bavaria, Germany) was produced using the lost wax technique. Following fabrication, the fit of the Co-Cr post-and-core was clinically evaluated. The restoration was carefully tried in the post space to verify passive seating and absence of rocking. The marginal adaptation at the core level was also visually inspected to ensure a precise fit with the remaining tooth structure.

**Figure 4:**
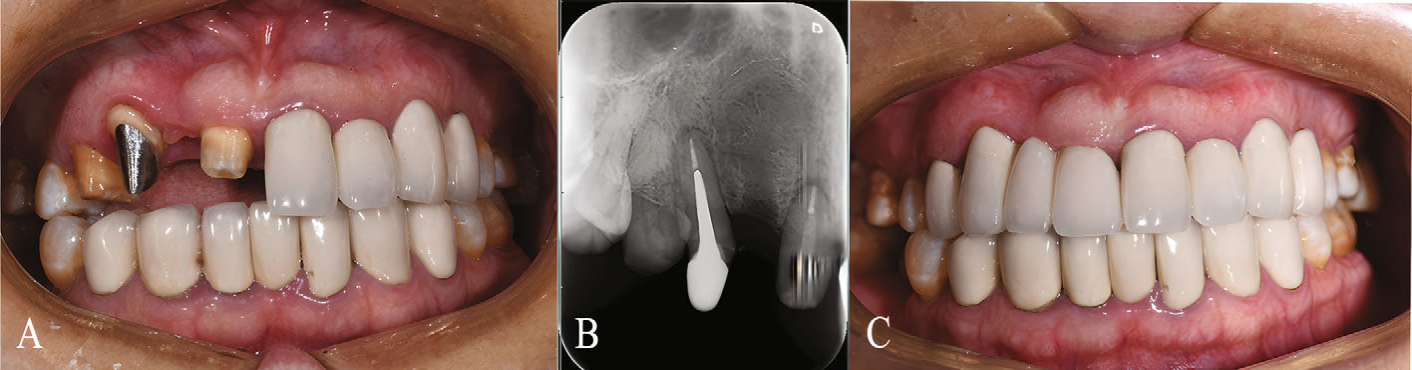
Final restoration. (A) Frontal view of the cemented post-and-core. (B) Periapical radiograph showing well-adapted seating of the cemented post-and-core. (C) Frontal view of the definitive cemented crown, demonstrating relatively improved aesthetic and functional outcomes in this case.

Following fitting and adaptation checks to ensure no further adjustments were required, the internal surface of the Co-Cr post-and-core was sandblasted (110 *μ*m alumina particles, 0.4 MPa, 10 sec). An eighth-generation bonding agent (Single Bond Universal, 3 M ESPE, St. Paul, MN, United States) was applied to the Co-Cr post-and-core and the prepared tooth structure, followed by resin cement (RelyX Ultimate, 3 M ESPE) according to the manufacturer's instructions to complete the luting procedure of the customized post-and-core restoration (Figure 4A,B).

Subsequently, the definitive tooth preparation for the crowns was completed. The abutments were then scanned with an IOS to capture the new emergence profiles. Based on this digital impression, a four-unit PFM fixed dental prosthesis spanning from tooth 11 to tooth 14 was designed and fabricated. The fabrication process involved milling a wax pattern, investing and casting the metal coping framework, and finally layering and firing the porcelain veneer (Figure 4C).

At the 3-month clinical follow-up, the patient reported satisfaction with both the function and aesthetics of the restoration. No signs or symptoms of periapical disease were observed.  Table 1 outlines the digital workflow checklist for customized post-and-core restorations utilizing scan posts.

## 3. Case Presentation 2

**Figure 5:**
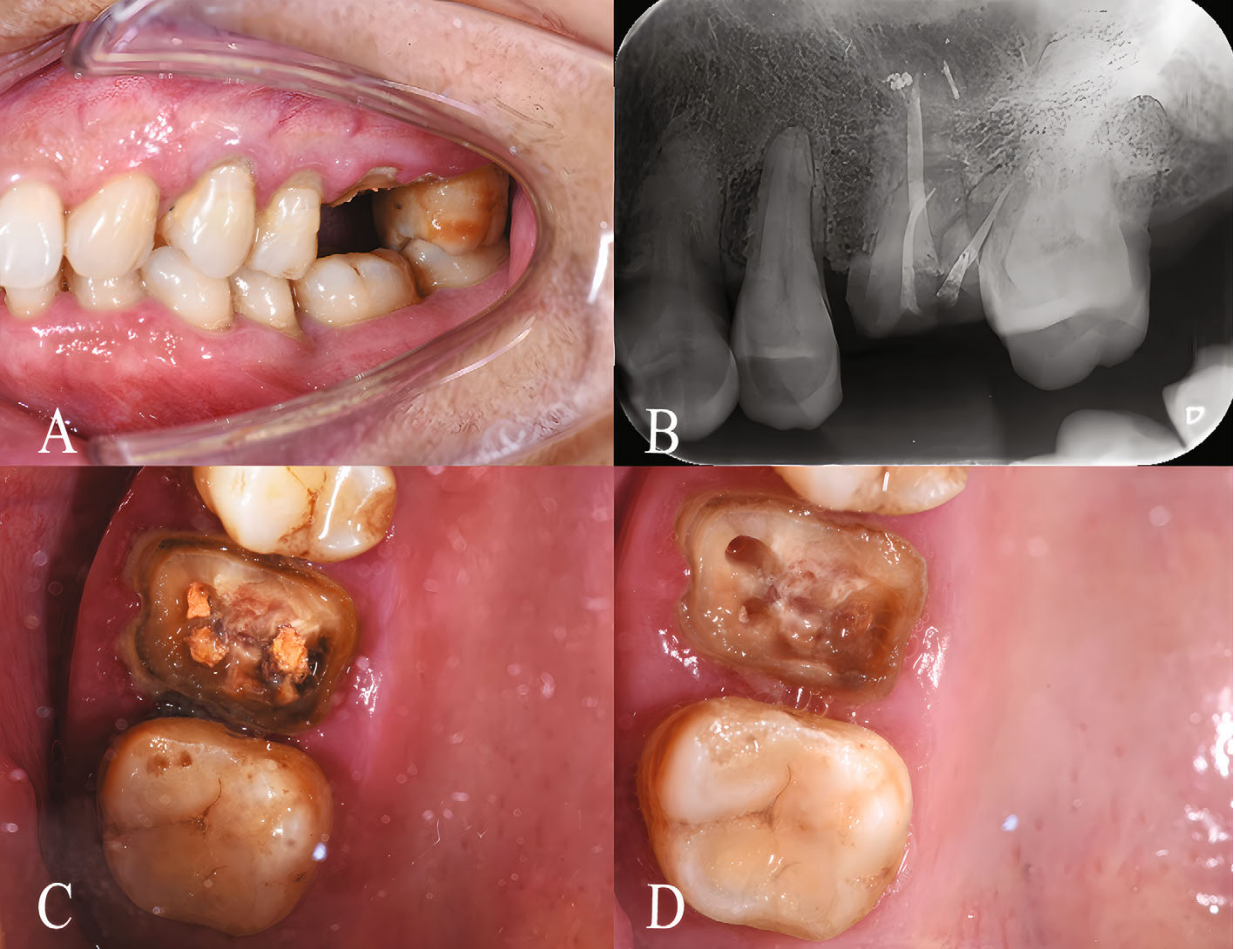
Fractured left maxillary first molar after endodontic treatment. (A) Buccal view. (B) Periapical radiograph. (C) Occlusal view post-endodontic treatment. (D) Occlusal view after post space preparation.

A 57-year-old female patient presented with a fractured left maxillary first molar, which had occurred 3 weeks prior during the mastication of hard food. Intraoral examination revealed that the fracture extended subgingivally to the gingival margin (Figure 5A). Radiographic evaluation confirmed that the tooth had previously undergone successful endodontic treatment (Figure 5B). The extensive fracture and resulting structural compromise were attributed primarily to the loss of tooth integrity following endodontic therapy, exacerbated by functional occlusal forces.

The primary goals of treatment were to restore the fractured molar to function while ensuring long-term biomechanical stability. Specific objectives included replacing missing tooth structure with a restoration capable of withstanding high occlusal loads and achieving a hermetic marginal seal to prevent secondary caries or endodontic failure. Aesthetic considerations, though secondary to function in this posterior region, were also integrated through the use of a zirconia crown.

Several treatment options were considered for the extensively damaged molar. A prefabricated fiber post was deemed unsuitable due to the significant loss of coronal structure and the high occlusal forces in the molar region. Alternatively, a customized Co-Cr split cast post-and-core was selected to provide superior mechanical retention and stress distribution within the root canal system. This approach allowed for better adaptation to the irregular canal morphology and enabled the creation of a ferrule design. The definitive restoration consisted of a zirconia crown, chosen for its excellent strength, durability, and biocompatibility. A fully digital workflow [11, 16] was employed, utilizing intraoral scanning and CAD/CAM technology to ensure precision and optimal fit of both the post-and-core and the final crown.

The mesio-buccal and palatal root canals of tooth 26 were sequentially prepared using #1 through #2 post drills (RelyX Fiber Post Drill, 3 M ESPE). To ensure sufficient length for the split cast post-and-core, a portion of the gutta-percha was removed while maintaining a 3–5-mm apical seal for periapical protection (Figure 5C,D).

**Figure 6:**
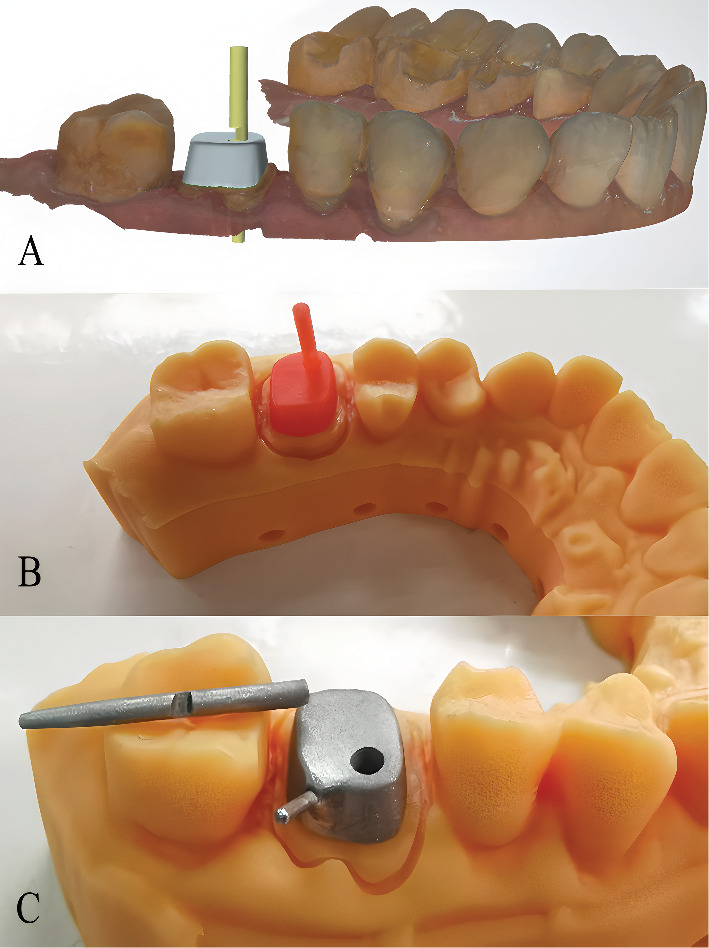
Computer-aided design and manufacturing process of the split cast post-and-core restoration. (A) CAD software visualization of the digitally designed split cast post-and-core, showing the precise adaptation to the root canal morphology and the separation plane between the segments. (B) Milled wax pattern of the post-and-core prior to investment casting, demonstrating the accurate reproduction of the digital design. (C) Final cobalt–chromium alloy split cast post-and-core after casting and finishing, ready for clinical try-in and cementation.

The mesio-buccal and palatal root canals were then scanned using TRIOS 3D IOS, both without and with the scan posts inside the post spaces. The digital files exported from the IOS were processed using CAD software (3Shape Dental System 2021, 3Shape), allowing for the design of a virtual post-and-core (Figure 6A). A wax pattern was subsequently milled using a CAM machine (ZENO 4030 M1+, imes-icore GmbH) (Figure 6B).

Finally, a Co-Cr alloy split cast post-and-core (15088-1, DFS-Diamon) was produced using the lost wax technique (Figure 6C).

**Figure 7:**
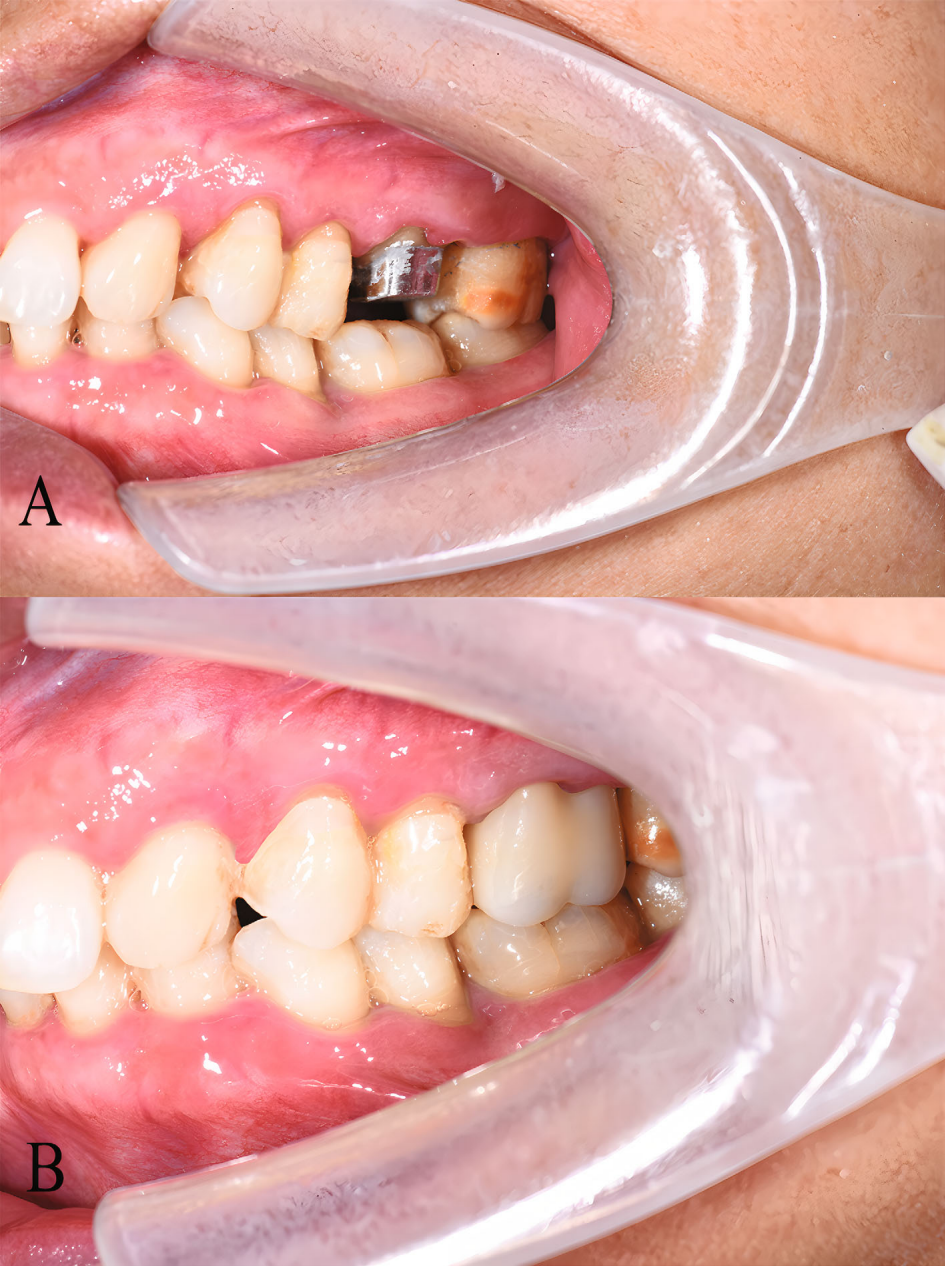
Cemented split post-and-core and final restoration. (A) Buccal view of the cemented split post-and-core. (B) Buccal view of the cemented crown.

The fit of the post-and-core was verified to ensure no adjustments were necessary, and the surface was cleaned in preparation for cementation with resin cement (RelyX Unicem, 3 M ESPE) (Figure 7A). After cementation, the tooth was scanned again with the IOS to capture the updated position of the post-and-core.

A zirconia crown (Multilayer, Aidite, Qinhuangdao, Hebei, China) was then milled, sandblasted (BASIC MASTER, Renfert GmbH, Hilzingen, Baden-Württemberg, Germany), and placed on the tooth (Figure 7B). The remaining restoration procedures were completed following standard clinical protocols.

At the 6-month clinical follow-up, the patient exhibited no signs of periapical infection, and the restoration remained functional and stable.

## 4. Discussion

This article discusses the application of digital impressions for post-and-core restorations in both anterior and posterior teeth. By designing a scan post that matches the shape of the post space, accurate data of the post space can be obtained.

While customized post-and-cores offer a precise fit in elliptical or tapered canals, cast metal posts may present aesthetic disadvantages. Advances in CAD-CAM technology have expanded the materials available for customized post-and-cores beyond traditional alloys. New materials, including zirconia, FRC, nanoceramic composite resins, and hybrid ceramics, offer improved options for both functionality and aesthetics [17].

The traditional technique using elastomeric impressions demands a high level of proficiency from practitioners. Even experienced clinicians may encounter challenges such as the formation of bubbles or voids in the impression [18], deformation of the impression material, or residue of the material remaining inside the root canal. Additionally, obtaining a complete impression for split post-and-core restorations, particularly in molars, can be particularly difficult. These issues can be effectively addressed through the use of digital impressions, which offer improved accuracy and ease of use.

By integrating IOS with CAD/CAM technology, post-and-cores can be fabricated to precisely match the root canal morphology. The process involves scanning the tooth with an IOS both without and with a scan post inside the post space. This approach allows for capturing the wide root canal entrance directly with the IOS, while the scan post helps accurately map the shaped canal wall in deeper sections. The findings of Zaki et al. [19] align closely with the clinical outcomes observed in our cases. Their in vitro study demonstrated that increasing post space depth significantly reduced scanning trueness, with a depth of 10 mm leading to clinically unacceptable discrepancies—a challenge also encountered in our deep canal cases [20]. While their results indicated that preparation width had no significant effect on trueness, our clinical experience supports the use of scan posts to mitigate depth-related inaccuracies, particularly in narrow and deep post spaces [21]. Unlike their model using the Panda P2 scanner without auxiliary devices, our application of custom scan posts with the TRIOS system provided additional geometric references, thereby improving data capture reliability in subgingival regions. This strategic use of scan posts offers a practical solution to the depth-related limitations highlighted in their study, enhancing the clinical viability of fully digital workflows for complex post-and-core restorations.

Even in cases with a wide root canal entrance, customized post-and-core restorations can achieve a precise fit within the post space. This results in a thin and evenly distributed cement layer, which helps minimize the risk of tooth fracture. Additionally, digital impressions eliminate the need for plaster models, significantly reducing material usage and chairside time. The digital impression technology also enables the storage of impression data on a computer, making it easy to copy, process, and reuse without restrictions.

Although traditional techniques may offer higher accuracy and retention rates compared to full digital and semidigital workflows, the retention and fit of posts using digital methods generally align with clinical guidelines [22].

An in vitro study has shown that direct application of IOS for reading post spaces is unreliable, particularly for relatively shallow root canals [23]. To address this issue, this article introduces the use of scan posts, which effectively capture data from narrow post spaces by utilizing predefined scan post data. This technology not only provides complete impression data but also overcomes the challenges associated with capturing impressions for split cast post-and-core restorations. Additionally it would be interesting in the future to test also the match of digital impressions with recently introduced features such as smartphone applications [24] and artificial intelligence software [25] in order to improve data and knowledge for daily clinical practice.

The decision to use custom-designed scan posts was based on both practical and technical considerations. Initially, the availability of manufacturer-provided scan posts compatible with our specific drill system was uncertain. Our custom posts were designed with a combined cylindrical and conical morphology. This geometric configuration offers greater seating tolerance and facilitates easier alignment within the post space compared to more complex shapes [15]. Furthermore, this simplified geometry enables seamless integration into the CAD software environment. By designing a virtual cylinder with matching dimensions in the software library, the scanned data of the post can be precisely aligned and utilized. This universal approach ensures compatibility with virtually any IOS and CAD system, eliminating dependence on proprietary closed ecosystems [26].

However, this study has several limitations that warrant discussion. First, the limited sample size of two clinical cases restricts the generalizability of our findings. Second, the relatively short follow-up period (3 and 6 months) necessitates further investigation into the long-term outcomes of this technique. Additionally, the absence of a post-cementation radiograph for Case 2 impedes immediate assessment of restoration adaptation in that case, underscoring the need for more rigorous documentation protocols in future research.

While the presented technique demonstrates clinical promise, it is imperative to acknowledge its inherent challenges and limitations, particularly when applied to deep and narrow root canals. A primary obstacle is the physical confinement of the canal itself, which can severely limit the angulation and movement of the IOS tip, potentially leading to incomplete data capture or stitching errors in the resulting digital impression [15]. Furthermore, the depth of the canal can cause attenuation of the scanning light, reducing signal accuracy in the apical portion and potentially introducing inaccuracies in the representation of the canal's true geometry. To mitigate these challenges, the use of a geometrically known scan post, as demonstrated in this report, is a critical strategy. This approach provides clear, unambiguous geometry for the scanner to capture, effectively acting as a radiographic marker and significantly enhancing the reliability of data acquisition in the most challenging regions of the post space.

Beyond data capture, other limitations must be considered. The risk of restoration misfit, though mitigated by CAD/CAM fabrication, persists. Dimensional inaccuracies can arise from any step in the digital chain, including polymerization shrinkage of the 3D-printed resin scan post, software design algorithms, or milling tolerances. Moreover, material compatibility presents a crucial consideration. The adhesive bonding of metallic post-and-cores (e.g., Co-Cr), as used in these cases, requires specific surface treatment protocols (e.g., sandblasting, application of metal primers) to achieve a durable bond with resin cements. This is in contrast to the bonding protocols for zirconia or FRC posts, indicating that the choice of material directly impacts the clinical protocol and potential long-term success.

Furthermore, the digital workflow allows for easy storage and management of impression data. It is crucial to note that the conclusions drawn from this case report are primarily based on clinical feasibility and short-term outcomes. Quantitative accuracy data and long-term follow-up imaging, which are necessary to substantiate claims regarding precision, are not within the scope of this clinical presentation. Nevertheless, preliminary support for the accuracy of this technique is provided by a recent in vitro investigation from our team [15], which found that the digital workflow with scan posts yielded superior accuracy compared to conventional half-digital techniques. Consequently, while the clinical results are promising, further rigorous clinical studies are warranted to conclusively determine the accuracy and long-term performance of this approach.

## 5. Conclusions

In conclusion, the integration of IOSs with CAD/CAM technology, facilitated by custom-designed scan posts, offers a precise and efficient digital workflow for fabricating post-and-core restorations in deep and narrow post spaces. This method overcomes limitations of traditional impressions, such as voids and material deformation, while ensuring a thin, uniform cement layer that enhances restoration stability and reduces fracture risk. Although the technique demonstrates clinical promise for both anterior and posterior teeth, further validation through laboratory studies and long-term clinical trials is essential to confirm its accuracy, reliability, and applicability across diverse clinical scenarios, including multi-rooted teeth and advanced restorative materials. Future research should also address challenges related to the retrieval of customized restorations and explore material-specific optimization.

## Figures and Tables

**Table 1:** Checklist of digital workflow procedures for customized post-and-core restorations using scan posts.

**Phase**	**Procedure step**	**Key details and considerations**
Preoperative assessment	Clinical and radiographic examination	Assess tooth condition, ferrule effect, periapical health, and root canal morphology.
	Treatment planning	Decide between prefabricated FRC post or customized post-and-core based on indication.

Tooth preparation	Removal of existing restorations/gutta–percha	Ensure a 3–5-mm apical seal of gutta-percha is maintained.
	Post space preparation	Use sequential drills corresponding to the desired post size.

Digital data capture	Scan post design and fabrication	3D print scan posts compatible with the prepared post space.
	Intraoral scanning (without scan post)	Capture the reference geometry of the prepared tooth and adjacent arches.
	Intraoral scanning (with scan post)	Capture the precise geometry of the deep and narrow post space.
	Bite registration	Record occlusal relationships digitally.

CAD/CAM fabrication	CAD design	Design the customized post-and-core restoration using the scanned data.
	Virtual cement gap setting	Set appropriate parameters for the cement space in the software.
	CAM milling	Mill the wax pattern or restoration from the chosen material.
	Casting/finishing	Use lost-wax technique for metal alloys; sinter for zirconia.

Restoration delivery	Try-in and fit check	Verify passive fit and adaptation of the post-and-core.
	Surface treatment	Sandblast internal surface of metal restorations for adhesion.
	Adhesive cementation	Use appropriate bonding agent and resin cement following protocols.
	Final prosthesis delivery	Cement the final crown/bridge after post-and-core placement.

Follow-up	Clinical evaluation	Assess function, aesthetics, and periapical health at scheduled intervals.

## Data Availability

The data that support the findings of this study are available from the corresponding author upon reasonable request.

## Nomenclature

FRC: fiber-reinforced composite
IOS: intraoral scanner
CAD/CAM: computer aided design/computer aided manufacturing
PFM: porcelain fused to metal
3D: three-dimensional
Co-Cr: cobalt–chromium
